# *Anais Brasileiros de Dermatologia*: metrics related to 2022 and position in the ranking of Dermatology journals^⋆^

**DOI:** 10.1016/j.abd.2023.08.002

**Published:** 2023-09-23

**Authors:** Sílvio Alencar Marques, Ana Maria Ferreira Roselino, Hiram Larangeira de Almeida Jr., Luciana P. Fernandes Abbade

**Affiliations:** a Department of Infectology, Dermatology, Diagnostic Imaging and Radiotherapy, Universidade Estadual Paulista, Botucatu, SP, Brazil; b Department of Dermatology, Universidade de São Paulo, Ribeirão Preto, SP, Brazil; c Department of Dermatology, Universidade Federal de Pelotas, Pelotas, RS, Brazil; d Department of Dermatology, Universidade Católica de Pelotas, Pelotas, RS, Brazil

Dear Associates,

This issue 99 (1) of the *Anais Brasileiros de Dermatologia* (ABD), contains a special article prepared by Miot et al.,^1^ aimed at the presentation and discussion of metrics, which inform the scientific community about the ranking of ABD when compared to other scientific journals in Medicine and Dermatology. Even taking into account possible biases, and academic differences among specialties, the editors of each scientific journal should reflect on future pathways and proposals to achieve a better ranking of the journal among its peers. Why is this important? In the first place, to attract articles with greater scientific rigor, not previsousely published, better documented and illustrated, with potential for calling international attention and citation. The authors will be rewarded by having their scientific productions recognized and cited, thanks to the scientific status of the journal in which they publish; and to be well evaluated by funding agencies and by the regulatory bodies of the *stricto sensu* Postgraduate Programs. Additionally, in the case of ABD, through the expression of good metrics and a good position in the ranking, authors and editors contribute to the maintenance of a virtuous circle of support and investment in scientific improvements and growth.

The metrics for 2022, released between June and July 2023, refer to the citation rates received by the journal during the year 2022 for articles published in the same journal in previous years.^1^ The three most frequently used metrics are the Scimago Journal Rank (SJR) which uses a broad database, in which ABD rose slightly (from 0.428 to 0.449), and rose to Quartile 2 (Q2) of journals in Dermatology. In the CiteScore, which uses a database that includes 26,500 journals, we moved from 2.3 to 2.4 and achieved the 60^th^ position among 133 journals and also Q2. The most acknowledged and valued metric, the Impact Factor (IF) of the Web of Sciences, considers citations for articles published in the two immediately previous years, uses data from 21,500 journals, and focuses, in the present case, on 70 journals related to Dermatology. Regarding 2022, the position of the ABD corresponded to the 50^th^ position, Q3, as a result of the reduction of its IF to 1.7 (compared to 2.13 in 2021). Miot et al. demonstrate that the percentage reduction in the IF occurred similarly for the set of Brazilian scientific journals that were evaluated as well as for the set of international Dermatology journals.

Knowledge of current metrics leads us to promote actions so that ABD can reach a more competitive ranking. The path to achieving this goal requires that ABD, the largest representative of the Brazilian Society of Dermatology (SBD, *Sociedade Brasileira de Dermatologia*), on the eve of completing 100 years of uninterrupted publication, be better appreciated and valued by its national peers. Greater visibility, reduction of the time between submission and publication, and better quality of documentation and review are desired. The articles, which dealt with national and international endemics, as observed in relation to cases of Mpox, dermatological side effects induced by COVID-19, and those related to anti-SARS-CoV-2 vaccines, had their publication expedited due to the imposed urgency, consistent with the above-mentioned proposals.^2^, ^3^, ^4^, ^5^

These are perennial commitments of the ABD Editorial Board and the SBD Board of Directors, and authors and readers are invited to join us in this task.

## Financial support

None declared.

## Authors' contributions

Silvio Alencar Marques: Approval of the final version of the manuscript; drafting and editing of the manuscript.

Ana Maria Roselino: Approval of the final version of the manuscript; drafting and editing of the manuscript.

Hiram Larangeira de Almeida Junior: Approval of the final version of the manuscript; drafting and editing of the manuscript.

Luciana P. Fernandes Abbade: Approval of the final version of the manuscript; drafting and editing of the manuscript.

## Conflicts of interest

None declared.
